# The Burden of Selected Chronic Non-Communicable Diseases and Their Risk Factors in Malawi: Nationwide STEPS Survey

**DOI:** 10.1371/journal.pone.0020316

**Published:** 2011-05-23

**Authors:** Kelias P. Msyamboza, Bagrey Ngwira, Titha Dzowela, Chimwemwe Mvula, Damson Kathyola, Anthony D. Harries, Cameron Bowie

**Affiliations:** 1 World Health Organisation, Malawi Country Office, Lilongwe, Malawi; 2 University of Malawi, College of Medicine, Community Health Department, Blantyre, Malawi; 3 Ministry of Health, Lilongwe, Malawi; 4 International Union Against Tuberculosis and Lung Disease, Paris, France; 5 Department of Infectious and Tropical Diseases, London School of Hygiene and Tropical Medicine, London, United Kingdom; Genentech Inc., United States of America

## Abstract

**Background:**

Chronic non-communicable diseases (NCDs) are becoming significant causes of morbidity and mortality, particularly in sub-Saharan African countries, although local, high-quality data to inform evidence-based policies are lacking.

**Objectives:**

To determine the magnitude of NCDs and their risk factors in Malawi.

**Methods:**

Using the WHO STEPwise approach to chronic disease risk factor surveillance, a population-based, nationwide cross-sectional survey was conducted between July and September 2009 on participants aged 25–64 years. Socio-demographic and behaviour risk factors were collected in Step 1. Physical anthropometric measurements and blood pressure were documented in Step 2. Blood cholesterol and fasting blood glucose were measured in Step 3.

**Results and Conclusion:**

A total of 5,206 adults (67% females) were surveyed. Tobacco smoking, alcohol drinking and raised blood pressure (BP) were more frequent in males than females, 25% vs 3%, 30% vs 4% and 37% vs 29%. Overweight, physical inactivity and raised cholesterol were more common in females than males, 28% vs 16%, 13% vs 6% and 11% vs 6%. Tobacco smoking was more common in rural than urban areas 11% vs 7%, and overweight and physical inactivity more common in urban than rural areas 39% vs 22% and 24% vs 9%, all with *p*<0.05. Overall (both sexes) prevalence of tobacco smoking, alcohol consumption, overweight and physical inactivity was 14%, 17%, 22%, 10% and prevalence of raised BP, fasting blood sugar and cholesterol was 33%, 6% and 9% respectively. These data could be useful in the formulation and advocacy of NCD policy and action plan in Malawi.

## Introduction

Previously considered as diseases of the affluent and a distraction from the business of prevention and control of communicable diseases [1], chronic non-communicable diseases (NCDs), in particular cardiovascular diseases (heart diseases and stroke), cancer, respiratory diseases and diabetes mellitus are increasingly becoming significant causes of morbidity and mortality in low- and middle- income (LMI) countries. Recent estimates suggest that NCDs are responsible for 60–64% of all deaths in these countries with 61% of deaths occurring in people younger than 70 years of age [2]–[6]. WHO projected that by 2015 NCDs will account for over 70% of all deaths globally with 80% of these deaths occurring in developing countries [2]. Developing countries are therefore having to cope with a double burden of communicable and non-communicable diseases, a situation that has been described as “a race against time’’ [1], [7]. NCDs together with HIV have resulted in many LMI countries failing to keep on track of reaching the Millennium Development Goal targets [8]. In sub-Saharan Africa (SSA), urbanisation, changing lifestyles, socio-cultural factors, poverty and poor maternal, foetal and infant nutrition, which forms the basis of the developmental origins of NCDs, are some of the drivers of this epidemic [1], [8]–[10].

Tobacco smoking, excessive alcohol consumption, physical inactivity, obesity, low fruit and vegetable intake are well known shared risk factors for the major NCDs. Assessing the epidemiological situation by identifying the distribution of risk factors among different population groups in a country is the first of the three WHO recommended planning steps for prevention and control of NCDs and their risk factors. In particular, the development of a national risk factor profile for NCDs provides key information required for planning prevention and control activities. Information on a risk factor profile could also help to predict the future burden of disease. This, in turn, would help to make a strong case for high-level advocacy and constitutes an evidence base for planning interventions at policy, environmental and health system levels. The second step is the formulation and adoption of an NCD policy and plan of action. A WHO generic NCD Policy and Plan of Action is available to help with this task [11], [12]. The third planning step is to identify policy implementation steps by which the policy and plan of action can be implemented, especially in the areas of health financing, legislation and regulations, advocacy, community-based interventions, and health services delivery [13]. The primary health care approach is the recommended most cost-effective approach for delivering NCD interventions [11], [14], [15]. Several publications have highlighted the need for local high quality epidemiological data on the burden of NCDs and their risk factors particularly in SSA where such data are scarce [14], [16]–[19]. Between July and September 2009, we conducted a nationwide cross-sectional survey using WHO NCD STEPS survey tools to determine the magnitude of NCDs and their risk factors in Malawi.

## Materials and Methods

### Ethics statement

Ethical approval was granted by the Malawi National Health Sciences Research and Ethics Committee. Written informed consent was obtained before participants were enrolled in the study using the WHO NCD STEPS survey consent form.

### Study design

This study was a nationwide population based cross-sectional survey designed according to a WHO STEPwise approach to chronic disease risk factor surveillance [20]. It is called STEPwise (STEPS) approach because data are collected in 3 steps; step 1 uses a questionnaire to collect demographic and lifestyle data; step 2 involves measurements of height, weight, blood pressure, waist and hip circumference; and step 3 uses laboratory (biochemistry) investigations.

### Sample size calculation

Sample size was calculated using the formula: 
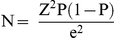


Where N =  sample size, Z =  level of confidence, P =  baseline level of the selected indicator and e =  margin of error, set at P = 0.50, Z = 1.96 (at 95% confidence interval), e = 0.05. The sample size was adjusted for design effect for complex sample design set at 1.50, age-sex estimates in the 25–64 age range (8 10-year intervals) and a non-response rate of 20%. The minimum calculated sample size was therefore multiplied by 1.5 and by 8, and then divided by 0.8 to adjust for design effect, age-sex estimates and non-response rate respectively. With these adjustments, the final sample was 5,760. It was assumed that the non-response rate would be high because participants may refuse blood testing and or not adhere to fasting, the latter being required for fasting blood glucose testing.

### Sampling of survey sites, households and eligible participants

Enumeration areas (EAs) were used as survey sites. Administratively, Malawi is divided into twenty-eight districts. In turn, each district is subdivided into smaller administrative units called traditional authorities (TAs). Each TA is sub-divided into EAs by the National Statistical Office (NSO). Enumeration areas are classified as urban or rural. Each EA has demographic data and a sketch map. The sketch map shows the EA boundaries, location of buildings, and other landmarks. The list of all EAs in Malawi from population and housing census conducted in June 2008 was obtained from the NSO. This list was used as a sampling frame for the random selection of EAs. According to the WHO NCD STEPS Survey manual Part 2 section 2 [20], in each EA 30–50 households could be selected and in each household only one eligible participant could be selected. We settled for 40 households per EA. Therefore to reach the sample size, the total number of EAs to be selected was 144 EAs (5760/40). The 144 EAs were randomly selected nationwide using the probability proportional to size (PPS) sampling method. In each EA, 40 households were randomly selected using systematic sampling method. Sampling interval was calculated by dividing the total number of households in the EA as given by the NSO by 40 (the number of households to be selected). At household level, only one eligible participant was selected using the Kish sampling method built-in personal digital assistants (PDAs). Households with no eligible participant were not replaced.

### Participant recruitment and data collection

Eligible participants were all adults aged 25–64 years. Participants were involved in the survey for two days: day one was for the questionnaire and body measurements and day two was for laboratory tests and blood pressure measurement. Formal written consent was obtained. Participants with abnormal physical or laboratory findings as defined below were counseled and referred to their nearest health facility for further action and follow up. Body measurements and laboratory tests were performed by nurses and clinical officers while enumerators conducted the interviews. A total of seven survey teams, each with 8 members were deployed to collect data over a period of 30 days between July and September 2009.

Step 1, the survey questionnaire, was programmed on the PDAs. It consisted of core (age, sex and education in years and current exposure to tobacco and alcohol, diet and physical activity), expanded (rural/urban setting, occupation, average household income) and optional (marital status, medical and health history, past history of smoking and alcohol consumption) variables. The medical and health history component included questions on medication, cigarette use, diabetes mellitus and hypertension. The English questionnaire was translated into two main local languages (*Chichewa* and *Tumbuka*).

Step 2 involved physical body measurements. Physical body measurements that were performed included blood pressure, height, weight, waist and hip circumference measurements. Blood pressure measurements were taken using battery powered digital blood pressure machines (Omron® M4-I). The participant was asked to sit on the chair and rest quietly for 15 minutes with his/her legs uncrossed. The left arm of the participant was then placed on the table with the palm facing upward.Three readings 3–5 minutes apart were then taken on the left arm. During the analysis the average of the last two readings was the final blood pressure reading. Waist circumference was measured using a tape-measure in centimeters, and the measurement was made in the mid-axillary line midway between the last rib and the superior iliac crest. Measurements were taken to the nearest 0.1 cm. Hip measurement was also made using a tape-measure placed horizontally at the point of maximum circumference over the buttocks. Measurements were taken to the nearest 0.1 cm. Height was measured with the participant standing upright against a wall on which a height mark was made. Measurements were taken with the participant in barefoot, standing with the back against the wall and head in the Frankfort position with heels together. The participant was asked to stretch to the fullest. After being appropriately positioned, the participant was asked to exhale and a mark with a white chalk was made to mark the height. The height was then measured in centimeters from the mark to the floor using the tape-measure. Measurements were taken to the nearest 0.1 cm. Weight measurements were taken on a pre-calibrated weighing scale (bathroom scale). The scales were calibrated daily using a known weight (1 kg packet of sugar). Participants were weighed dressed in light clothing and barefoot. Measurements were taken to the nearest 0.1 kg.

Step 3 involved laboratory tests. On the first day of the survey after step 1 and step 2, participants were asked to starve overnight. Consenting participants were asked not to consume any food except for clear water after taking supper/dinner of that day until the survey team came again in morning of the following day (day 2). People converged at the agreed place in their community where finger prick blood samples for biochemistry tests were taken. Those that complied with advice (starving overnight) were eligible for finger prick blood sample collection. Total cholesterol and fasting blood glucose were measured using Accutrend® *Plus* machines.

### Data management

Data were collected electronically using PDAs (HP) programmed with WHO e-STEPS software. There were two sets of PDAs, one set for Step 1 (questionnaire) and Step 2 (body measurements) and the other set for Step 3 (biochemical measures). A total of 50 PDAs were used. Data on the PDAs were downloaded into the computer installed with WHO NCD STEPS software. The files of each participant (questionnaire, body measurements, biochemistry tests and Kish data) were then merged using the participant identity (PID) number cross-checked with participant name, EA number or village/township name and other particulars where necessary. After merging, common variables in the dataset were matched and inconsistencies were corrected.

Data were weighted by calculating sample weights for all records using the probability of selection at each stage of sampling. Thus, for each participant his/her weight was calculated by multiplying the probability of EA selection, the probability of household selection, the probability of selection within household and age-sex distribution of the population in Malawi. The participant's weight was equal to the inverse of this product. Data analysis was conducted in Epi Info, version 3.5.1 (Centres for Disease Control and Prevention, Atlanta, Ga). Confidence intervals (CI) for proportion were calculated using the formula p = p±C√p(p-1)/n where p is the given proportion whose CI needs to be calculated, C =  is the coefficient, at 95%CI C = 1.96, n =  number of participants. The results were statistically significant if there was no overlap between two CIs of comparing groups (males vs females, urban vs rural, study population vs general population).

### Definitions

Raised blood pressure was defined as a diastolic blood pressure of 90 mmHg or more or systolic blood pressure of 140 mmHg or more or currently on medication for high blood pressure (documented in the health booklet). Diastolic blood pressure ≥110 mmHg or systolic ≥180 mmHg was considered as severe high blood pressure. Raised fasting blood glucose was defined as blood glucose level ≥7.0 mmol/L or currently on medication for diabetes mellitus (documented in the health booklet). Raised total cholesterol was defined as cholesterol level ≥5.0 mmol/L. Overweight was defined as body mass index (BMI) ≥25.0 and obesity as BMI ≥30.0. Excessive or harmful use of alcohol was defined as consumption of 5 or more for men, 4 or more for women standard units per day for three or more days per week.

## Results

### Characteristics of participants enrolled in the survey

**Figure 1:**
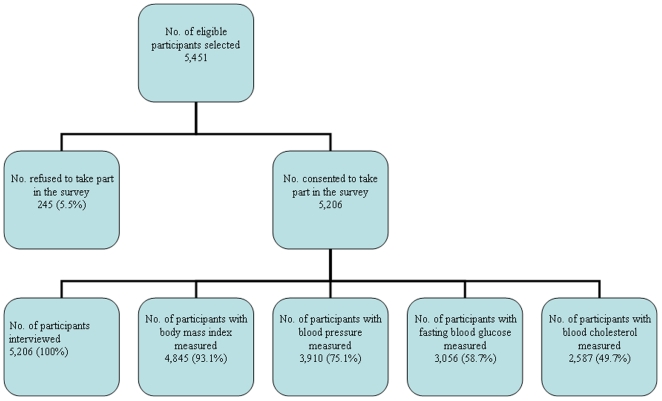
Flow diagram.

Of the 144 EAs that were selected, 143 were reached and data were collected. Only one EA was not reached and data were not collected because permission was not granted, it being a high security area. A total of 5,451 eligible adults were selected and approached to participate in the survey. Of these, 245 (5.5%) refused while 5,206 (95.5%) consented to take part in the survey. Of the 5,206 participants that took part in the survey, about two thirds (67.5%) were females, 87.4% were from rural areas and about one in four (24.6%) had no formal education. Blood pressure, fasting blood sugar and total cholesterol were measured in 75.1%, 58.7% and 49.7% respectively of the 5,206 participants (figure 1 and table 1).

**Table 1:** Characteristics of participants enrolled in Malawi NCD STEPS survey compared to National Statistics Office (NSO) 2008 population figures.

	NCD STEPS Survey participants 2009						NSO population figures 2008					
	Total		Male		Female		Total		Male		Female	
	n	%	n	%	n	%	n	%	n	%	n	%
**Sex**	5,206	100	1,690	32.5*	3,516	67.5	4,050,801	100.0	1,993,578	49.2	2,319,214	50.8
**Age (years):**												
25–34	2,335	44.9	719	42.5*	1,616	46.0*	1,930,523	47.7	947,720	47.5	982,821	47.8
35–44	1,321	25.4	459	27.2	862	24.5	1,064,561	26.3	542,189	27.2	522,372	25.4
45–54	902	17.2	296	17.5	604	17.2	612,824	15.1	294,063	14.8	318,761	15.5
55–64	650	12.5	216	12.8	434	12.3	442,893	10.9	209,624	10.5	233,269	11.3
25–64	5,206	100.0	1,690	100.0	3,516	100.0	4,050,801	100	1,993,578	100.0	2,057,233	100.0
**Marital status:§**												
Never married	161	3.1	91	5.4	70	2.0	249,046	5.4	176,871	7.9	72,175	3.1
Currently married	3,819	73.5	1,475	87.4	2,344	66.8	3,661,729	80.3	1,960,672	87.4	1,701,037	73.4
Separated/divorced	754	14.5	99	5.9	655	18.6	299,871	6.6	67,924	3.1	231,947	10.0
Widowed	464	8.9	22	1.2	442	12.6	350,784	7.7	36,729	1.6	314,055	13.5
Total	5,198	100.0	1,687	100.0	3,511	100.0	4,561,430	100	2,242,216	100.0	2,319,214	100.0
**Education:#**												
None	1,285	24.7	237	14.0	1,048	29.8	-	-	-	-	-	-
Standard 1-5	1,807	34.8	558	33.0	1,249	35.6	-	-	-	-	-	-
Standard 6-8	1,391	26.7	539	31.9	852	24.2	-	-	-	-	-	-
Secondary and above	720	13.8	355	21.1	365	10.4	-	-	-	-	-	-
Total	5,203	100.0	1,689	100.0	3,514	100.0	-	-	-	-	-	-

§Marital status NSO data includes those aged ≥65, *statistically significant, p<0.05, survey male participants vs NSO male population figures, #No comparable NSO data on education, n =  number in the group, CI =  confidence interval.

### Magnitude of selected NCDs and their risk factors

Tobacco smoking, alcohol drinking (any amount) and excessive alcohol drinking were more common in men than women (25.9% vs 2.9%, 30.1% vs 4.2%, 19.0% vs 2.3% respectively, all *p*<0.05). Overall (both sexes) the prevalences of these risk factors were 14.1%, 16.9% and 7.7% respectively. Hand-rolled cigarettes and home brew alcohol were the commonest types (over 90%) of cigarettes and alcohol drink taken. Overweight, obesity and physical inactivity were more frequent in women than men (28.1% vs 16.1%, 7.3% vs 2.0% and 12.6% vs 6.3% respectively, all *p*<0.05%). For both sexes combined, the prevalences were 21.9%, 4.6% and 9.5% respectively. Raised blood pressure or currently on blood pressure medication was more common in men than women (37.2% vs 29.2% *p*<0.05). Raised total cholesterol was more frequent in women than men (11.0% vs 6.3% *p*<0.05). There were no significant differences between men and women in the prevalence of raised fasting blood glucose or currently on medication (6.5% vs 4.7%), and having three or more risk factors for NCD (17.6% vs 15.4%). Overall, the prevalence of raised blood pressure or currently on medication, fasting blood glucose or currently on medication, raised total cholesterol, and three or more risk factors were 32.9%, 5.6%, 8.7% and 16.5% respectively. On average, participants ate fruits and vegetables 2.0 and 5.6 days per week respectively.

Overweight, obesity and physical inactivity were more common in urban than rural areas (38.6% vs 21.9%, 13.6% vs 4.9%, 24.1% vs 8.7% respectively all *p*<0.05). Tobacco smoking was more common in rural than urban areas (10.9% vs 6.6% *p*<0.05). There were no significant differences by urban/rural area in the prevalence of raised blood pressure or currently on medication, raised fasting blood glucose or currently on medication, alcohol drinkers and three or more risk factors.

Majority (93.3%) of people with raised blood pressure were not aware that they had such medical problem. Table 2 summarises the magnitude of the selected NCDs and their risk factors.

**Table 2:** Magnitude of selected NCDs and their risks factors in Malawi: July to September 2009.

	Total		Male			Female			Urban			Rural		
	n	%	n	%	95%CI	n	%	95%CI	n	%	95%CI	n	%	95%CI
Raised BP or currently on medication	3,910	32.9	1,183	37.2*	34.3 – 41.6	2,727	29.2	26.8 – 30.9	408	27.9	23.5–32.3	3502	32.0	30.5–33.6
Raised FBS or currently on medication	3,056	5.6	911	6.5	2.8 – 10.3	2,144	4.7	2.5 – 7.1	371	7.4	4.7–10.1	2685	9.2	8.1–10.3
Raised cholesterol	2,587	8.7	775	6.3	5.2 – 8.9	1,812	11.0*	9.7 – 12.9	-	-	-	-	-	-
Overweight (BM≥25 kg/m^2^)	4,845	21.9	1,664	16.1	14.1 – 18.0	3,181	28.1*	25.9 – 30.2	603	38.6*	34.7–42.5	4242	21.9	20.7–23.1
Obesity BM≥30 kg/m^2^	4,845	4.6	1,664	2.0	1.3 – 2.6	3,181	7.3*	6.3 – 8.4	603	13.6*	10.9–16.7	4242	4.4	3.8–5.0
Tobacco smokers	5,206	14.1	1,690	25.9*	23.3 – 28.5	3,526	2.9	2.1 – 3.8	655	6.6	4.7–8.5	4551	10.9*	10.0–11.8
Smokeless tobacco users	5,206	3.5	1,690	1.9	1.3–2.9	3,526	5.0*	4.0–6.0	-	-	-	-	-	-
Alcohol drinkers	5,206	16.9	1,690	30.1*	27.3 – 33.4	3,526	4.2	3.2 – 5.1	655	13.4	10.8–16.0	4551	12.5	11.5–13.5
Excessive alcohol drinkers	5206	7.7	1,690	19.0*	16.5–21.5	3,526	2.3	1.6–3.1	-	-	-	-	-	-
Physical inactivity	4,057	9.5	1,355	6.3	4.8 – 7.8	2,702	12.6*	10.6 – 14.7	519	24.1*	20.4–27.8	3538	8.7	7.8–9.6
Three or more NCD risk factors	2,842	16.5	940	17.6	15.2 – 20.8	1,902	15.4	13.3 – 17.4	281	22.6	17.7–27.5	2561	15.5	14.1–16.9

BP =  Blood Pressure, CI = Confidence interval, FBS =  Fasting blood glucose, n =  number of participants in the group, *statistically significant, p<0.05; male vs female, urban vs rural.

## Discussion

This study demonstrated that chronic non-communicable diseases and their risk factors are major public health problems in Malawi with at least one in four men smoking tobacco, one in five drinking alcohol excessively and at least one in four women being overweight. A third (32.9%) of the adult population aged 25–64 years had raised blood pressure or were on anti-hypertensive medication, 5.6% had raised fasting blood glucose or were on medication and 8.7% had raised cholesterol. The burden of NCDs in Malawi was demonstrated previously using a burden of disease methodology that estimated the prevalence of diabetes mellitus, ischaemic heart disease, and stroke for the adult population aged 30–69 years to be 13.6%, 4.4% and 6.1% respectively [21]. While our result for fasting blood glucose seems low compared to this previous estimate, the level of hypertension is much higher than expected. Automatic blood pressure device used this survey could have influenced the results although *Omron M4* machines were validated and passed the validation process [22], [23].

These findings confirm reports that NCDs are major public health problems in sub-Saharan Africa where they may account for 20% of all deaths [24], with the burden projected to rise to 40% by 2015 [25]. The majority of people with raised blood pressure (>90%) did not know that they had such medical problem and this is consistent with findings from other studies in sub-Saharan Africa [26]. High blood pressure is the leading cause of stroke in Africa [26]. The prevailing high health facility utilization rates and the existing outreach clinic programmes in the country could be used as an opportunity for screening high blood pressure. The primary health approach to raise awareness, screen, diagnose, treat and follow up people with NCDs is recommended [11], [15]. The potential value of screening people with tuberculosis for diabetes mellitus and people with diabetes for tuberculosis [27]–[31] and hypertension [32] is highlighted.

Non-communicable diseases and their risk factors were gender related, with tobacco smoking, alcohol consumption and raised blood pressure being more frequent in males than females whereas overweight, obesity and raised cholesterol were more frequent in females than males. In SSA, being overweight/obese could be perceived as being rich in males or sexually attractive in females [9]. This emphasises the need for taking into account gender related socio-cultural issues in NCD health promotion interventions. In the present study, two thirds of participants were females. However, it is unlikely that this had an influence on the results for women because data were weighted (standardised) for age and sex to national population. The over representation of females was not by study design as eligible participants were randomly selected using the Kish sampling method built-in the PDAs after entering their name, sex and age. It is uncertain whether the refusals/non-availability had an influence on the results. Refusals were relatively small (245) (Figure 1) with no differences between males and females and no replacements were made. Specifically, males aged 25–34 years were the ones that were underrepresented based on National Statistical Office Population figures 2008 (42.5% vs 47.5%, p<0.05). The under representation of men in this age group may be due to some being away from home at the time of the survey. It is not known if this group had different survey characteristics. All the other age groups were representative of the national population (Table 1).

Technical faults of the biochemistry machines (Accutrend *plus*®) particularly for cholesterol led to only half of consented and available participants being tested. Two machines were sent back to the manufacturer (Roche). It is uncertain whether failure to measure blood pressure, fasting blood glucose and cholesterol in all the participants who consented had an influence on the results. The curtailment of testing affected men, women and all age groups equally. Based on number of participants and their ages, the available results for each measurement were standardised for age and sex to the national population. The findings presented in this paper are therefore age-sex adjusted estimates. Nevertheless this community based study captured blood pressure measurement, blood cholesterol and fasting blood glucose tests for over 2,500 adults. Challenges caused by blood pressure and biochemistry measuring devices have been highlighted.

The association between NCD risk factors and urban/rural area of residence with overweight/obesity and physical inactivity being more frequent in urban than rural areas has also been reported in other studies in SSA [1], [8], [9]. Tobacco smoking was more common in rural than urban areas. Tobacco is widely grown in Malawi and is readily available in rural areas. However hypertension and raised fasting blood sugar were just as common in rural as in urban areas and therefore interventions should target both groups of people.

Taxation is one the strategies for controlling tobacco and alcohol use [11]. However, in Malawi, this strategy may have limited or no effect because most people (over 90%) smoke hand-rolled (self-made) cigarettes and drink home brewed alcohol. Modest alcohol consumption is inversely associated with risk of cardiovascular diseases and therefore the focus would be on prevention of harmful alcohol consumption particularly among males.

Vegetable intake was not a problem in this study population. People ate vegetables on average 6 days a week. But fruit intake was not sufficient, on average 2 days a week (recommended 3 days or more per week). However, the study was done in the dry season when fruits are scarce. In the rainy season when fruits particularly mangoes are plentiful the figure is likely to be higher.

In conclusion, this study presents evidence on the magnitude of NCDs and their risk factors, gender and urban/rural differences in Malawi, a poor country in central-southern Africa. These data could be useful in the formulation and advocacy of NCD policy and plan of action in Malawi.
